# Analysis of Phthalates and Alternative Plasticizers in Gloves by Gas Chromatography–Mass Spectrometry and Liquid Chromatography–UV Detection: A Comparative Study

**DOI:** 10.3390/toxics9090200

**Published:** 2021-08-28

**Authors:** Kelly Poitou, Tiphaine Rogez-Florent, Marie Lecoeur, Cécile Danel, Romain Regnault, Philippe Vérité, Christelle Monteil, Catherine Foulon

**Affiliations:** 1 Normandie Univ UNIROUEN, UNICAEN, ABTE, 76000 Rouen, France; kelly.poitou1@univ-rouen.fr (K.P.); philippe.verite@univ-rouen.fr (P.V.); christelle.monteil@univ-rouen.fr (C.M.); 2 Univ. Lille, CHU Lille, ULR 7365-GRITA-Groupe de Recherche sur les formes Injectables et Technologies Associées, 59000 Lille, France; marie.lecoeur@univ-lille.fr (M.L.); cecile.danel@univ-lille.fr (C.D.); romain.regnault@univ-lille.fr (R.R.); catherine.foulon@univ-lille.fr (C.F.)

**Keywords:** phthalates, alternative plasticizers, gas chromatography–mass spectrometry (GC–MS), liquid chromatography–diode array detector (HPLC–DAD), gloves

## Abstract

Gloves represent an essential feature for hand protection because it is a requirement in the professional framework to comply with both hand hygiene standards and the principles of good laboratory practice. Despite their wide use, there is a knowledge gap regarding their composition, including phthalates. The purpose of the present study was to develop two orthogonal methods, GC–MS and HPLC–DAD, for the screening of plasticizers in gloves. Performances of these two methods were compared in terms of ease of use, number of analyzed plasticizers, and sample preparation. The two methods were validated and applied for the identification and quantification of plasticizers in ten gloves made with different materials (vinyl, nitrile, latex, and neoprene). Results revealed the presence of three main ones: DEHP, DEHT, and DINP. Additionally, the contents of plasticizers were extremely variable, depending on the glove material. As expected, the results point out a predominant use of plasticizers in vinyl gloves with an amount that should be of concern. While DEHP is classified as a toxic substance for reproduction 1B, it was, however, quantified in the ten different glove samples studied. This study provides new data regarding the plasticizers’ content in protective gloves, which could be useful for risk assessment.

## 1. Introduction

In 2007, a report from the Occupational Safety and Health Administration estimated the number of workers wearing gloves for chemical protection in the United States at 5.8 million [1]. Gloves are widely used for medical applications (e.g., healthcare, surgery) and other non-medical applications in laboratories and industries. With the extensive use of protective gloves worldwide, the number of end-users is expected to be highly significant. Different types of protective gloves are employed, such as vinyl, latex, neoprene, or nitrile. Beyond these categories, protective gloves can also be classified according to the benefit of their use, i.e., as personal protective equipment (PPE), for protection from hazardous substances, as well as medical devices (MD) in the health sector. For such use, gloves such as vinyl gloves must comply with both PPE and MD regulations. In some applications, plasticizers such as phthalates (PAEs) are added to the polymer in order to confer characteristics of flexibility, softness, durability, and malleability. Among plasticizers identified, some are of great concern because human health effects involving carcinogenicity, endocrine disruption, and fertility impairment have been described [2,3,4]. Due to their reprotoxic effects, several phthalates are now classified as toxic substances for reproduction 1B according to the European Regulation No 1272/2008 on classification, labeling, and packaging of substances and mixtures including benzyl butyl phthalate (BBP), dibutyl phthalate (DBP), diisobutyl phthalate (DIBP), and bis-(2-ethylhexyl)phthalate (DEHP) [5]. Limitations on use with restrictions for toys and childcare articles, food contact plastics as well as medical devices have been decided. These successive regulations on phthalates coupled with rising concern about their human health effects have encouraged the use of alternative plasticizers with lower toxicity (e.g., long-chain phthalates, adipates, terephthalates) [6,7,8].

To our knowledge, there are few studies evaluating plasticizers in protective gloves despite their widespread use. Most studies on the subject were conducted to evaluate the permeability capability of organic solvents or in regard to the specification limits established for food contact materials [9,10,11]. These studies have reported the presence of plasticizers, such as BBP, DBP, DIBP, and DEHP. Moreover, Tsumura et al. [12] identified the migration of DEHP and other plasticizers by food contact with PVC gloves during meal preparation in Japan. They have shown that the DEHP level accounted for 21–41% (*w*/*w*) by mass in the PVC gloves. 

As plasticizers are not covalently bound to the polymer matrix, they can be released into the surrounding environment and can affect the human body [13,14,15,16,17,18,19,20]. Among products manufactured with those plasticizers, gloves appear to be an indubitable source of dermal exposure in the workplace. Therefore, studies are needed to identify and quantify which plasticizers are contained in gloves. Hence, it is important to develop efficient analytical tools to determine plasticizers content in gloves. It will provide new elements to establish whether gloves tend to follow the current legislation about plasticizers.

Gas chromatography (GC) coupled with flame ionization (FID) or mass spectrometry (MS) detection and liquid chromatography (LC) coupled with MS or with UV detection have been reported for the analysis of plasticizers in several types of matrices such as cosmetics and medical devices [4,11,13,17,21,22]. GC–MS and liquid chromatography coupled with diode array detector (HPLC–DAD) were chosen because they are orthogonal techniques in terms of separation performance and preparation of samples. 

The first aim of this work was to develop and validate two efficient orthogonal methods, i.e., GC–MS and HPLC–DAD, for the simultaneous analysis of the selected plasticizers. The validated methods were then applied to the identification and quantification of plasticizers in protective gloves after a simple extraction step with an organic solvent. The results obtained with the two developed methods are compared and discussed, considering the performance and the limits of each one.

## 2. Materials and Methods

### 2.1. Chemicals

The eleven phthalates and the two non-phthalates studied plasticizers are listed in Table 1. Benzyl butyl phthalate (BBP; ≥98%), dibutyl phthalate (DBP; ≥99%), di-cyclohexyl phthalate (DCHP; ≥99%), di(2-ethylhexyl) phthalate (DEHP; ≥99.5%), di-(2-ethylhexyl) terephthalate (DEHT; ≥97%), diethyl phthalate (DEP; ≥99.5%), diethyl phthalate-3,4,5,6-d_4_ (DEP-d_4_, IS; ≥98%), diisobutyl phthalate (DIBP; ≥99%), diisononyl phthalate (DINP; ≥99%), dimethyl phthalate (DMP; ≥99%), di-n-octyl phthalate (DNOP; ≥98%), diphenyl phthalate (DphP; ≥99%), dipropyl phthalate (DPP; ≥98%), and tri-octyl trimellitate (TOTM; ≥99%) were purchased from Sigma-Aldrich (Saint-Quentin Fallavier, France). HPLC-grade solvents were used. Acetone, dichloromethane, and acetonitrile were purchased from VWR (Fontenay-sous-Bois, France). Ultra-pure water was supplied by a Milli-Q purification system from Millipore (St Quentin en Yvelines, France).

### 2.2. Plasticizers

Since the nature and the quantity of plasticizers are unknown in gloves, our challenge was to develop an exhaustive analytical method adapted for the determination of a large scale of concentrations from trace levels, for the subsidiary plasticizers, to high contents for the main ones. According to the context of substitution, regulated and alternatives plasticizers were simultaneously assessed in the present study. The plasticizers selected for the present work included two extensively used PAEs (DMP and DEP), five phthalates classified as reproductive toxicant category 1 H360 (DIBP, DBP, BBP, DCHP, and DEHP), two plasticizers suspected to be reproductive toxicants category 2 H361 (DPP and TOTM), and three alternative plasticizers reflecting current trends in response to restriction policies (DEHT, DINP, DNOP, and DphP). 

### 2.3. Gloves Samples

Neoprene, nitrile, latex, and vinyl gloves used at the Faculty of Pharmacy from the University of Rouen (Rouen, France), the University of Lille (Lille France), and the Rouen University Hospital (Rouen, France) were studied in this work. Ten samples were chosen from different suppliers of gloves to consider the variability in the manufacturing procedure. The distribution of sampling was the following: one neoprene-, one nitrile-, three latex-, and five vinyl-based gloves.

### 2.4. Gas Chromatography Method

#### 2.4.1. GC–MS Instrumentation and Analysis Conditions

All analyses were performed using an Agilent 7890 gas chromatography system coupled with an Agilent 5975C mass spectrometer equipped with an electron impact (EI) ion source and a single quadrupole (Agilent Technologies, Santa Clara, CA, USA). Chromatographic separation was achieved with a cross-linked poly 5% diphenyl/95% dimethylsiloxane HP-5-MS capillary column (30 m × 0.25 mm i.d. × 0.25 µm film thickness) from Agilent. The oven temperature was initially set at 100 °C held for 2 min, then ramped at 30 °C min^−1^ to 200 °C held for 1 min, then 10 °C min^−1^ to 250 °C held for 6.5 min, and then 40 °C min^−1^ to 280 °C held for 16 min. One microliter of each sample was injected in splitless mode using an Agilent 7693A autosampler. The solvent delay was set at 5.0 min and the run time at 35 min. The injection port and the transfer line were both heated at 280 °C. The source and the single quadripole of the mass spectrometer temperatures were set at 230 °C and 150 °C, respectively. The carrier gas was helium at a flow rate of 1.0 mL min^−1^, and ionization with an inert EI ion source was operated at 70 eV. The software MSD Chemstation E.02.02.1431(Agilent Tech. Inc., Les Ulis, France) was used to control the instrument and was used to collect data.

#### 2.4.2. Choice of *m*/*z* Ions for SIM Acquisition

The acquisition was performed on full-scan (*m*/*z* = 50–650) and on selected ion monitoring (SIM) mode, for which acquisition parameters are detailed in Table 2 with a dwell time of 100 ms per analyte. Sequential analysis of ions was performed using three time windows (groups 1–3). On SIM mode, two different ions were monitored for each plasticizer. They were determined according to the literature (NIST library) and after a previous injection using the full-scan mode. The retention time (RT) was determined for each analyte after the injection of a standard solution. As DINP is a mixture of structural isomers composed of variable branches C9 chained alcohol isomers, it is thus eluted as a broad mass peak. For its integration, the choice was made to select a defined portion of the multi-isomeric peak (from 18.3 to 19.5 min) to allow reproducibility.

#### 2.4.3. Stock and Standard Solutions

For the preparation and the storage of the standard solutions, only glass materials were used to prevent the external contribution of plasticizers from plastics. Cleaned glass materials were rinsed twice with the containing organic solvent. All individual stock solutions were prepared at a concentration of 10 g L^−1^ in acetone for DMP, DEP, DEP-d_4_ (IS), DPP, DIBP, DBP, DCHP, DEHP, DEHT, DINP, DNOP, and TOTM, or in dichloromethane for DphP and DCHP. The working solutions of the thirteen plasticizers and the IS (individual and mixture solutions) were daily prepared in acetone by diluting the stock solutions to achieve the targeted concentrations. All solutions were stored at 4 °C. For method validation, calibration standards were prepared in acetone with concentrations ranging from 0.05 to 0.5 mg L^−1^ for DMP, DEP, DPP, DIBP, DphP, and DBP; from 0.5 to 6 mg L^−1^ for BBP; from 0.3 to 3 mg L^−1^ for DEHP and DEHT; from 15 to 135 mg L^−1^ for DINP; and from 5 to 60 mg L^−1^ for TOTM. In both cases, four validation standards were prepared at 0.06, 0.15, 0.35, and 0.50 mg L^−1^ concentration levels for DMP, DEP, DPP, DIBP, DphP, and DBP; 0.75, 2.5, 4.5, and 6 for BBP; 0.40, 1.25, 2.25, and 3.00 mg L^−1^ for DEHP and DEHT; 15, 45, 90, and 120 mg L^−1^ for DINP; and 25, 45, and 60 mg L^−1^ for TOTM.

#### 2.4.4. Extraction Protocol

Each glove was cut into small pieces, and samples were weighted (around 30 mg) in a 5 mL glass tube. This mass was chosen for a miniaturized procedure for sample preparation. For the extraction of the plasticizers, samples were covered with 2.0 mL of acetone. Sealed glass tubes were then put into an ultrasonic bath at room temperature and sonicated for 30 min. Following the extraction step, all samples were spiked with the IS: 10 µL of a 1 mg L^−1^ IS solution (final concentration of 0.01 mg L^−1^) were added to 990 µL of the extract, and the final solution was vortexed prior to its injection in the GC–MS system. During the extraction step, a blank sample underwent a similar extraction procedure.

### 2.5. High-Performance Liquid Chromatography Method

#### 2.5.1. HPLC–DAD Instrumentation and Analysis Conditions

Chromatographic analyses were performed using a Waters system (Milfors, MA, USA) equipped with a gradient quaternary 600E metering pump, an online degasser apparatus, a 717 autosampler, and a 996 photodiode array detector. Data were collected and processed on a computer running with Empower software (version 2) from Waters. Separations were carried out on a reversed-phase Kinetex C18 (100 × 4.6 mm i.d., 2.6 μm) column (Phenomenex, Le Pecq, France). A C18 trap column (LiChrospher^®^100 RP18: 100 × 2.1 mm i.d., 5 μm, Interchim, Montluçon, France) was connected between the solvent mixing chamber and the injector of the HPLC apparatus. A gradient separation mode was used to separate the analytes, using acetonitrile (A) and water (B). The elution gradient performed at 0.8 mL min^−1^ was the following: (1) 0–10 min, linear increase from 50% to 100% of solvent A; (2) 10–25 min, 100% of solvent A; (3) 25–26 min, linear decrease to 50% of solvent A; and (4) 26–35 min, 50% of solvent A. The column was thermostated at 30 °C, and the injection volume was 20 μL. Detection was performed at 225 nm except for DEHT, which was detected at 240 nm.

#### 2.5.2. Stock and Standard Solutions

Stock and standards solutions for HPLC experiments were prepared in glass vessels using the same experimental precautions as in GC. Stock solutions of the thirteen plasticizers at 10 g L^−1^ were prepared in acetonitrile and stored at 4 °C. A working solution containing 100 mg L^−1^ of DINP and 10 mg L^−1^ of DMP, DEP, DPP, DphP, BBP, DIBP, DBP, DCHP, DEHP, DEHT, DNOP, and TOTM was prepared in an acetonitrile/H_2_O-50/50 (*v*/*v*) mixture by dilution of stock solutions. It was further diluted to obtain a solution at 2 mg L^−1^ for DINP and 0.4 mg L^−1^ for the other plasticizers used during the method development.

For method validation, two distinct series of calibration and validation standards were prepared. The first series, containing DMP, DEP, DPP, DphP, BBP, DBP, DCHP, DEHP, DEHT, DNOP, and TOTM, was prepared in acetonitrile/H_2_O-50/50 (*v*/*v*) with concentrations ranging from 0.05 to 1 mg L^−1^. The second series, prepared in the same solvent, contained DIBP and DINP at concentrations ranging from 0.05 to 1 mg L^−1^ and from 0.5 to 10 mg L^−1^, respectively. In both cases, four validation standards were prepared: the concentration levels of validation standards were equal to 0.15, 0.35, 0.5, and 1.0 mg L^−1^ for all plasticizers, except for DINP, for which they were 1.5, 3.5, 5, and 10 mg L^−1^.

#### 2.5.3. Extraction Protocol

As for GC analysis, around 30 mg of each glove were cut into small pieces and introduced in a glass tube before the addition of 2 mL of acetone. Extraction was then performed for 30 min at room temperature by immersion of the sealed tube in an ultrasonic bath. A volume of 1.5 mL of extracts was then evaporated at room temperature under nitrogen flow. The residues were dissolved in 1 mL of acetonitrile and finally diluted in order to obtain acetonitrile/H_2_O-50/50 (*v*/*v*) solutions that plasticizers concentration is included in the range of the calibration standard. During the extraction step, a blank sample underwent a similar extraction procedure.

### 2.6. Method Validation

The validation of the method was performed according to the validation guidelines proposed by the French Society of Pharmaceutical Sciences and Technologies—SFSTP [23]. In GC–MS, the validation was performed simultaneously using standard solutions containing the eleven plasticizers (DMP, DEP, DPP, DIBP, DBP, BBP, DEHP, DphP, DEHT, DINP, and TOTM) and IS. In HPLC–DAD, two distinct validations were performed: the first was dedicated to DMP, DEP, DPP, DphP, BBP, DBP, DCHP, DEHP, DEHT, DNOP, and TOTM analysis; and the second for DIBP and DINP. Hence, in each case, the validation of the method was carried out on three consecutive days to estimate the prediction errors. Each day, six calibration standards (CS) and four validation standards (VS; *n* = 3) were prepared and analyzed. Moreover, blank extracts were daily prepared and analyzed (*n* = 4; CAL0). They consisted of acetone spiked with the IS and acetonitrile/H_2_O-50/50 (*v*/*v*) solutions for GC–MS and HPLC–DAD, respectively, and were obtained from 2 mL acetone samples having undergone extraction procedures used for each method. A blank extract without IS was also analyzed in GC–MS (*n* = 4; CAL00). Finally, specificity, response function, linearity, trueness, and precision (repeatability and intermediate precision) were studied. The acceptance criteria for precision (relative standard deviation; RSD) and trueness (recovery) were fixed at 15% and in the 85–115% range, respectively. Lastly, accuracy profiles were assessed using NeoLiCy^®^ software (version: 1.8.2.2), considering the ±15% acceptance limits at a risk of 5%. They were used to determine the lower limits of quantification (LLOQ). In addition, carryover was estimated by injecting the blank samples after a mixture of the analytes (concentrations equal to the upper limit of quantification) containing or not the internal standard, according to the method evaluated. The stability of the stock solutions and working solutions was assessed by comparing freshly prepared solutions to solutions that have been stored at 4 °C for two months. 

## 3. Results 

### 3.1. Gas Chromatography–Mass Spectrometry

#### 3.1.1. Optimization of the Method

**Figure 1:**
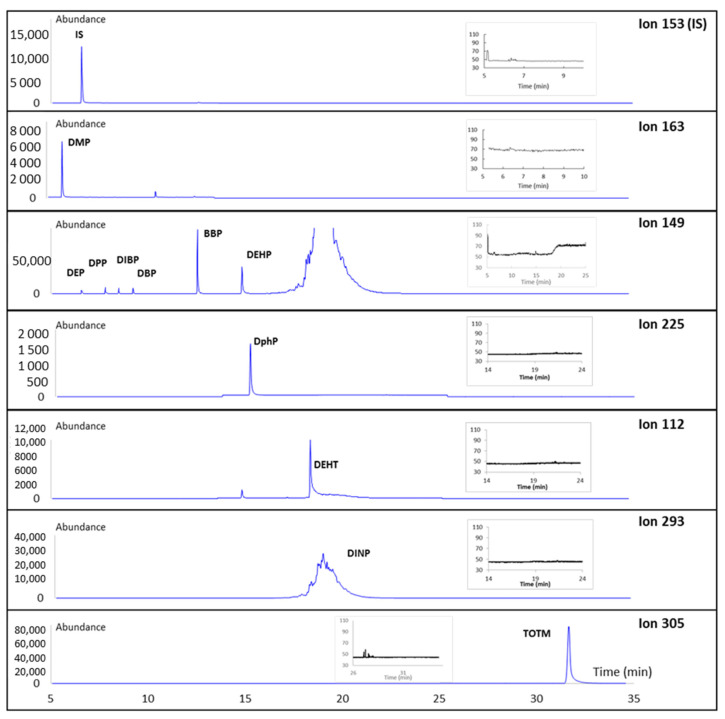
GC–MS chromatograms of plasticizers investigated.

The optimization of the GC–MS chromatographic conditions for the simultaneous determination of the thirteen targeted plasticizers was based on the GC–MS method developed by Gimeno et al. [7] and used for the identification and quantification of fourteen phthalates and five non-phthalate plasticizers in PVC medical devices. Several adjustments were made to optimize the method for the plasticizers of the study, including eleven phthalates (BBP, DBP, DCHP, DEHP, DEP, DIBP, DINP, DMP, DNOP, DphP, and DPP) and two non-phthalate plasticizers (DEHT and TOTM). Among the selected phthalates, it should be noted that DphP, which was not studied in the work of Gimeno et al., was selected for our study because of its interesting status as an alternative plasticizer. The successive changes made on the GC oven program allowed the total separation of nine plasticizers; DNOP, DEHT, and DINP were partially co-eluted. The identification and the quantification of the targeted plasticizers were assessed using SIM mode. For DEHT and DINP, a specific ion was selected to identify and quantify each of these analytes, even in a mixture with other analytes. For DNOP, no specific *m*/*z* ratio was identified. Hence, DNOP was discarded from the GC–MS method. DCHP did not have an acceptable chromatographic profile to include it in our study. For this reason, eleven plasticizers can be studied in GC–MS using acquisition parameters summarized in Table 2. Figure 1 shows the chromatogram of the standard solution mixture at the estimated LLOQ. 

SIM chromatograms of the first validation standard (blue) and a blank solution (CAL00; black). Acquisition performed on three time windows: see Table 2.

#### 3.1.2. Validation of the Method

Firstly, the specificity of the method with respect to DMP, DEP, DPP, DIBP, DBP, BBP, DEHP, DphP, DEHT, DINP, and TOTM was assessed by comparing chromatograms obtained for the blank extracts (CAL00), a blank extract spiked with the IS at the concentration used for sample analysis (CAL0), and a blank extract spiked with IS and the analytes at a concentration corresponding to the first VS. As illustrated in Figure 1, the absence of interfering components demonstrated the specificity of the method.

**Figure 2:**
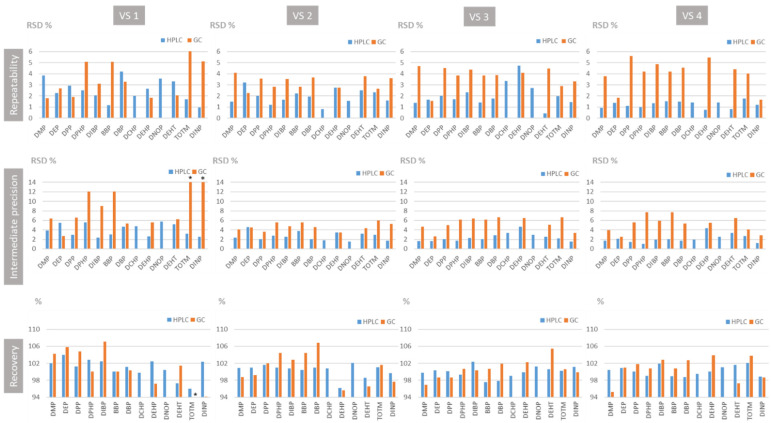
Repeatability, intermediate precision and recovery results of the GC–MS and HPLC–DAD method validation for the analysis of plasticizers. * RSD of intermediate precision and recovery were not in accordance with the acceptance criteria.

Response functions expressing the peak area ratio (analyte/IS) versus analyte concentration were established from six calibration standards: the best adjustments were obtained using least-square weighted (1/X) linear regression for DMP, DEP, DPP, DBP, DEHP, DEHT, and DIBP; least-square quadratic regression for DphP, BBP, and DINP; and least-square linear regression for TOTM. Obtained results are summarized in Table 3. The back-calculated concentrations of the calibration standards were within ±15% of the nominal values, even for the LLOQ. The validity of the model was attested by evaluation of the linearity of the method: whatever the analyte, the introduced concentrations and the back-calculated one can be expressed by linear models (R^2^ > 0.97) with slopes close to the unit and y-intercept that can be considered to be equal to zero (Student’s test, with α = 5%). Repeatability and intermediate precision were expressed as the relative standard deviation (RSD) at each concentration level. Trueness was evaluated by comparing nominal and back-calculated concentrations and was expressed as recovery. As shown in Figure 2, repeatability, i.e., intra-day precision, ranged from 1.56% to 5.45%. Concerning the intermediate precision (inter-day precision), RSD values were between 2.52% and 12.07%, except for the lowest validation standard (VS1) of DINP and TOTM that exhibited an RSD of 32.05%. Recovery varied between 94.1% and 107%. Hence, except for DINP and TOTM at the lowest concentration, the trueness and precision results are in accordance with set acceptance criteria. These results were used to calculate the upper and the lower confidence limits for validation standards required to establish the accuracy profiles (Supplementary Figure S1). 

Whatever the concentration level, the tolerance interval (β = 5%) is included in the acceptance limits set at ±15% for DEP, DBP, DEHT, DEHP, and TOTM. The lower limits of quantification (LLOQ) are equal to 0.020 mg L^−1^ for DEP and DBP, to 0.40 mg L^−1^ for DEHT and DEHP, and to 25.0 mg L^−1^ for TOTM. For DMP, DPP, DphP, BBP, DINP, and DIBP, the accuracy profiles exhibit a tolerance interval excluded from the acceptance limits for the lowest concentrations. Hence, for each of these analytes, the LLOQ can be calculated by taking the intersection point between the acceptability limit and the corresponding tolerance interval; they are equal to 0.040 mg L^−1^ for DMP and DPP, 0.060 mg L^−1^ for DphP, 2.40 mg L^−1^ for BBP, 40.14 mg L^−1^ for DINP, and 0.05 mg L^−1^ for DIBP.

Carryover was estimated by injecting blank extract after a mixture of the analytes (concentration equal to the upper limit of quantification). The peaks areas obtained for the blank sample following the highest concentration standard were less than 10% of the LLOQ for the analytes and less than 5% for the internal standard. These results are in accordance with the EMA criterion. None of the analytes was detected on the chromatogram, reflecting the absence of carryover. Analyte stock solutions appeared stable for two months of storage at 4 °C as assessed by a deviation from the initial concentration within ±15%.

### 3.2. High Performance Liquid Chromatography–UV Detection

#### 3.2.1. Optimization of the Method

##### Optimization of the Elution Gradient

**Figure 3:**
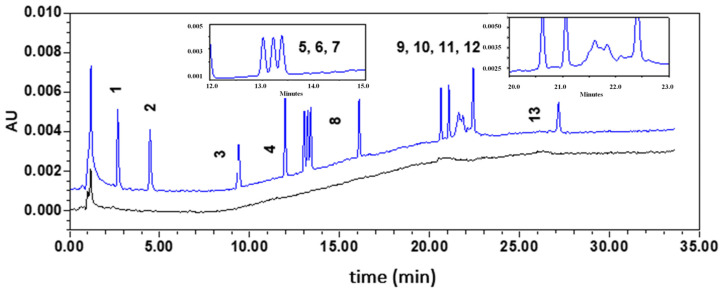
HPLC–DAD chromatograms of plasticizers investigated. Chromatograms of the first validation standard (blue) and a blank solution (CAL0; black). Compounds were eluted between 2.8 and 27.3 min according to the following order: (1) DMP, (2) DEP, (3) DPP, (4) DphP, (5) BBP, (6) DIBP, (7) DBP, (8) DCHP, (9) DEHP, (10) DNOP, (11) DINP, (12) DEHT, and (13) TOTM. Inserted chromatograms correspond to a zoom of the 12–16 min and 20–23 min periods.

Firstly, performances of a biphenyl (250 × 4.6 mm; 5 µm) and a Kinetex C18 (100 × 4.6 mm i.d., 2.6 μm; Phenomenex) columns were evaluated using acetonitrile (A) and water (B) at 0.8 mL min^−1^ and the following elution gradient: (1) 0–20 min, linear increase from 60% to 100% of solvent A; (2) 20–35 min, 100% of solvent A; (3) 35–36 min, linear decrease to 60% of solvent A; and (4) 36–46 min, 50% of solvent A. A mixture of thirteen plasticizers at 0.4 mg L^−1^, except DINP at 2 mg L^−1^, was injected in each column thermostated at 30 °C. While the biphenyl phase led to eleven peaks, of which only four were separated (Rs > 1.5), the C18 phase permitted to detect thirteen peaks, of which seven were totally resolved. Moreover, peak efficiencies were greater using the C18 phase (twice factor). The Kinetex C18 column was then selected to further optimize the separation conditions by changing the initial acetonitrile content and the gradient shape. Hence, optimal separation was obtained using the following elution gradient: (1) 0–10 min, linear increase from 50% to 100% of solvent A; (2) 10–25 min, 100% of solvent A; (3) 25–26 min, linear decrease to 50% of solvent A; and (4) 26–35 min, 50% of solvent A. Figure 3 depicts the resulting chromatogram (blue line). As shown in the insert, despite gradient modification, DIBP and DBP were partially resolved (Rs = 1.09). Hence, if DIBP and DBP are both present in gloves, their quantification will not be possible using the HPLC–DAD method. Moreover, DINP, which corresponds to a complex mixture of C9 ester isomers, was co-eluted with DEHT. In mixture with DEHT, quantification of DINP could be performed at its maximal absorbance wavelength, i.e., 225 nm, using the first part of the recording signal (from 21.20 to 21.68 min) to avoid errors resulting from the residual signal of DEHT measured at 225 nm. For DEHT, quantification at 240 nm can be envisaged as the absorbance of DINP is negligible at this wavelength. 

##### Setting-Up a Trap Column

Injection of an acetonitrile/H_2_O-50/50 (*v*/*v*) solution (composition equivalent to the mobile phase at the beginning of the gradient) in the optimal experimental conditions exhibited a residual peak for DEHT, whose intensity was greater each day just after system switching on. After a deep investigation, we concluded that this contamination resulted from the LC-system, probably from the leaching of DEHT contained in the HPLC tubings. This phenomenon was avoided by connecting a C18 trap column (100 × 2.1 mm i.d., 5 μm) between the solvent mixing chamber and the injector of the HPLC apparatus, as suggested by Descat et al. [24] for DEHP analysis. No change in the chromatographic parameters was observed. 

#### 3.2.2. Validation of the Method

**Figure 4:**
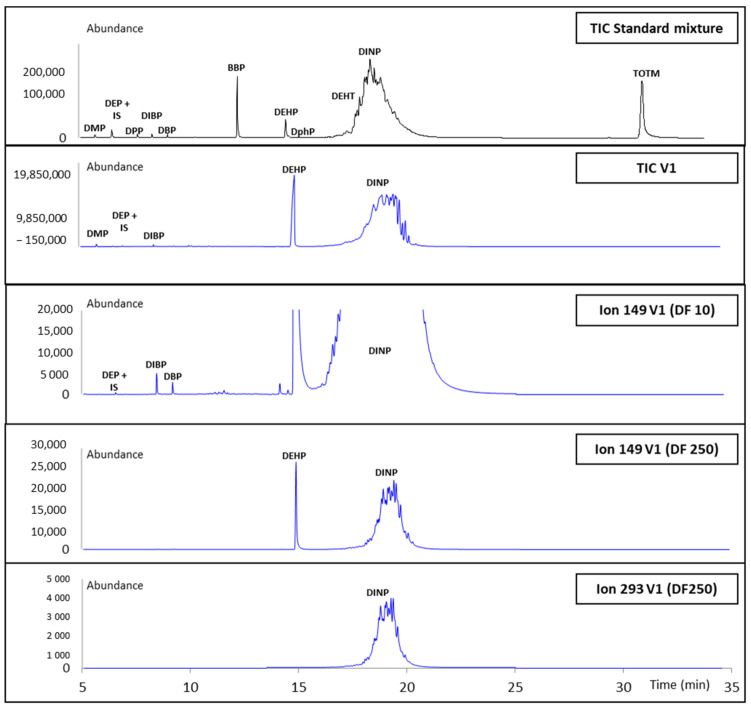
GC–MS chromatograms of the calibration standard SC2 (black) and a solution prepared from the V1 glove extraction (blue) at different dilution factors (DF 10 and 250). DF: dilution factor.

Firstly, the specificity of the method with respect to DMP, DEP, DPP, DpHP, BBP, DIBP, DBP, DCHP, DEHP, DINP, DEHT, DNOP, and TOTM was assessed by comparing chromatograms obtained for the blank extracts (CAL0) and a blank extract spiked with the analytes at the concentration corresponding to the first VS (Figure 4). The absence of interfering components demonstrated the specificity of the method. 

According to the separative performances of the method (partial resolution of DIBP and DBP) and coelution of DINP and DEHT, two series of six calibration standards were prepared: the first series contained DMP, DEP, DPP, DphP, BBP, DBP, DCHP, DEHP, DEHT, DNOP, and TOTM, while the second contained DIBP and DINP. They were used to assess the response functions expressing the peak area versus concentration using least-square linear regression for all compounds except for DEP, DPP, and DINP, for which least-square weighted (1/X) linear regression was used. Obtained results are summarized in Table 3. The back-calculated concentrations of the calibration standards were within ±15% of the nominal values, even for the LLOQ. Concerning DINP, it is noteworthy that the response function was established considering the area of the peaks of the compounds included in the complex mixture eluted alone, i.e., separate from DEHT, eluted between 21.20 and 21.68 min (time range of 0.48 min from the peak start). It could be used for its quantification alone or in a mixture with DEHT. As for the GC–MS method, the validity of the model was attested by evaluation of the linearity of the method (Student’s test, α = 5%) (Table 3). As shown in Figure 2, RSD expressing repeatability ranged from 0.43% to 4.71%. Concerning the intermediate precision, RSD values were between 1.21% and 5.98%. Recovery varied between 96.0% and 104%. These trueness and precision results, in accordance with set acceptance criteria, were used to calculate the upper and the lower confidence limits for validation standards required to establish the accuracy profiles (Supplementary Figure S2). Whatever the concentration level, the tolerance interval (β = 5%) was included in the acceptance limits set at ±15%. Hence, the lower limit of quantification (LLOQ) was equal to 0.05 mg L^−1^ for all compounds except for DINP, which exhibited an LLOQ equal to 0.5 mg L^−1^. As in GC–MS, no carryover was highlighted, and analyte stock solutions appeared stable for two months of storage at 4 °C.

### 3.3. Application to Gloves Analysis

Several strategies are described in the literature for the extraction of plasticizers from PVC medical devices [25]. Simplicity, rapidity, and efficiency of solvent extraction led us to select this approach as in the study of Chao et al., which showed the extraction of different plasticizers from vinyl, nitrile, and neoprene gloves [11]. Around 30 mg of each glove (corresponding to an area of 1.5 cm^2^) were cut into small pieces and introduced in a glass tube before the addition of the extraction solvent. The sealed tube was then immersed in an ultrasonic bath to perform the extraction. As extraction yield was shown to depend on the solvent used, especially on its polarity, different solvents were evaluated, i.e., cyclohexane, dichloromethane, and acetone. Indeed, it is difficult to choose a solvent based on the literature, as data available concern only extraction of a restricted number of plasticizers (DBP, DEHP, DEHT, and TOTM from PVC medical devices or gloves) and show that a solvent suitable for one plasticizer may not be convenient for another [11,26]. Preliminary experiments have shown that vinyl and neoprene gloves melt in dichloromethane and that extractions performed in acetone lead to a greater number of peaks in GC–MS. Hence, acetone was selected as an extraction solvent. The influence of the volume of acetone (from 2 to 4 mL) and duration of the extraction (from 10 to 60 min) using ultrasounds were evaluated at room temperature. It is noteworthy that the solvent volume must permit complete immersion of the glove sample and avoid reaching the solubility limit of the plasticizer during extraction. The use of 2 mL of acetone combined with the application of ultrasounds for 30 min was finally selected to obtain extracts in a short time, with higher plasticizers concentrations for better detection. 

This protocol was applied to ten different glove samples: five vinyl gloves (V1–V5), three latex gloves (L1–L3), one nitrile glove (N1), and one neoprene glove (Neo1), in order to check the presence of plasticizers and to determine their contents. Each glove sample was extracted six times independently; the first three extracts were analyzed by GC–MS and the others by HPLC–DAD, applying a separate sample preparation protocol for each method (see experimental section). For GC–MS analysis, the extract was spiked with IS (dilution negligible), leading to a sample that can be either directly analyzed (to detect a great number of plasticizers) or after a dilution step in order to obtain a response in the range suitable for their quantification (dilution factor between 2 and 2000). For HPLC–DAD analysis, as solubility in acetone and in the mobile phase used at the beginning of the gradient (ACN/H_2_O-50/50 (*v*/*v*) mixture) differs, a solvent exchange was performed to avoid precipitation of analytes in the system: a 1.5 mL volume of the extract was evaporated at room temperature under nitrogen flow. The residue was dissolved in 1 mL of ACN. This solution was first diluted using a minimal dilution factor (two-fold factor for nitrile and latex gloves; 10-fold factor for neoprene gloves, and 500-fold for vinyl gloves, respectively) in order to obtain an ACN/H_2_O-50/50 (*v*/*v*) solution which plasticizers concentration was just under the solubility limit to detect and identify a great number of them. Secondly, to quantify them, an appropriate dilution is necessary to include their concentration in the calibration range.

In HPLC–DAD, according to the dilution performed, among the thirteen plasticizers searched, none of them was detected in nitrile and neoprene gloves. For latex gloves, DIBP and DBP were observed in the extracts but not quantified. In vinyl gloves, high quantities of plasticizers led us to apply high dilution factors, resulting in the quantification of DEHP, DINP, and/or DEHT, exclusively. It is noteworthy that the two additional plasticizers that can be quantified in HPLC–DAD with respect to GC–MS (DCHP and DNOP) were finally not detected in the extracts.

Concerning GC–MS, while this method exhibits higher limits of detection with respect to HPLC–DAD, as a negligible dilution is performed before the first analysis, it allows to highlight the presence of at least four plasticizers in all gloves and to quantify them systematically.

**Figure 5:**
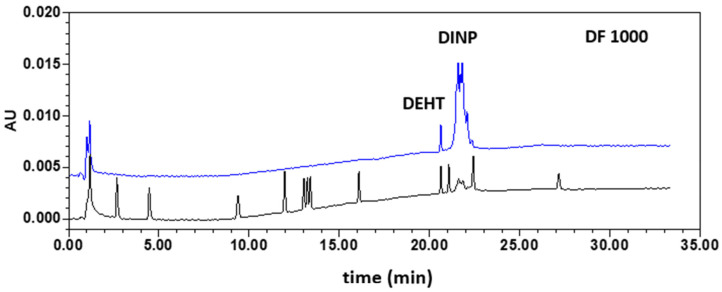
HPLC–DAD chromatograms (λ = 225 nm) of the calibration standard SC2 (black) and a solution prepared from the V1 glove at a dilution factor of 1000 (blue). DF—dilution factor.

**Figure 6:**
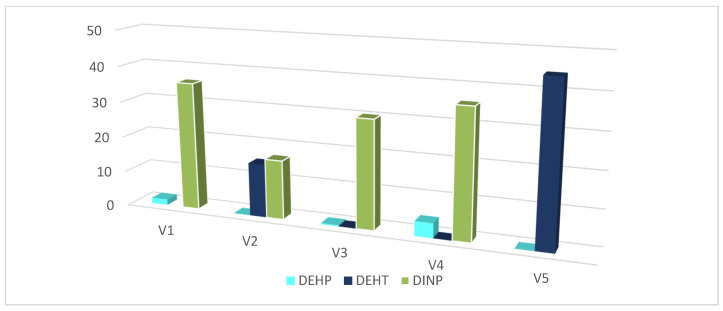
Contents of the majority plasticizers in the five vinyl gloves studied, determined by GC–MS.

Typical chromatograms for a vinyl (V1) glove sample obtained using GC–MS and HPLC–DAD methods are shown in Figure 4 and Figure 5. Only seven out of the thirteen plasticizers studied have been identified in glove samples (DMP, DEP, DIBP, DBP, DEHP, DEHT, and DINP). DINP, DIBP, and DBP have been detected in all samples. As expected, according to the literature [11], a total amount of plasticizers of less than 0.2% (*w*/*w*) has been detected in nitrile, neoprene, and latex gloves (data not shown). Results obtained for the analysis of plasticizers in the five vinyl gloves are presented in Table 4. The total amount of plasticizers in vinyl gloves were ranging from 30% to 44% (*w*/*w*), with trace levels of minority plasticizers. Whereas the contents of majority plasticizers determined by GC–MS and HPLC–DAD are matching, the minority plasticizers have not been quantified by HPLC–DAD. Figure 6 shows the distribution of the majority of plasticizers in the five vinyl gloves analyzed in GC–MS. The repartition of the three majority plasticizers, i.e., DEHP, DINP, and DEHT, in vinyl gloves is very disparate. DEHP was quantified in all the vinyl gloves, with contents ranging from 0.00062% (*w*/*w*) to 4.10% (*w*/*w*), while DINP was only present in four vinyl gloves with a content ranging from 16.5% (*w*/*w*) to 35.8% (*w*/*w*). DEHT was mostly quantified in the V5 glove (44.4% (*w*/*w*)). Since the extraction validation was made without glove standards, plasticizer contents were considered only as estimations.

## 4. Discussion

Human exposure can occur not only through ingestion, inhalation, and parenteral route but also through dermal absorption [13,14,15,16,17,18,19,20]. Dermal exposure is often considered a negligible route of exposure to plasticizers in comparison to other routes. With regard to the results of quantification of the plasticizers in gloves which are described in this study, it seems appropriate to review the dermal route in light of these new elements. Indeed, the results highlight high contents for DEHP, DEHT, and DINP in vinyl gloves. Hence, supplementary data are needed because, to date, skin exposure to plasticizers with gloves remains poorly described. These results should be then completed with additional studies on the migration of plasticizers with the purpose to provide new data for appropriate evaluation. This will reflect in a more relevant way of the real exposure since the migration of plasticizers from gloves is worrying due to direct dermal contact and can contribute to greater concern with possible toxic effects in the human body. Consequently, current legislations seem inappropriate because the recommendations do not include gloves despite the fact that they are manufactured with plasticizers. The limited information available about the content of plasticizers in gloves made it a sensitive issue, especially because it was not considered a topic of research in their own right.

## 5. Conclusions

In this study, the analytical performances of two orthogonal methods, i.e., GC–MS and HPLC–DAD, were compared for the identification and the quantification of plasticizers in protective gloves. After optimization, both analytical methods were validated according to the total error approach. In GC–MS, while higher limits of detection and quantification were obtained with respect to HPLC–DAD, a lower dilution factor applied to glove extracts before their analysis allowed to quantify the minority plasticizers. This method can be applied to a larger sample of gloves.

This study has shown that the content of plasticizers in protective gloves is variable depending on the type of glove (latex, nitrile, neoprene, or vinyl). As expected, results point out a predominant use of plasticizers in vinyl gloves with an amount that should be of concern. While DEHP is classified as a toxic substance for reproduction 1B, it was quantified in the ten different glove samples studied. The results of this study demonstrate that workers wearing gloves for chemical protection are faced with an additional source of exposure to phthalates. To reduce human exposure to some phthalates, this work could initiate the first steps to a glove selection process with consideration for the plasticizers’ content.

## Figures and Tables

**Table 1:** Plasticizers investigated in this study.

Compound	Acronym	Molecular Formula	Molecular Mass	CAS Number
			(g/mol)	
**Phthalates**				
Benzyl butyl phthalate	BBP	C_19_H_20_O_4_	312.36	85-68-7
Dibutyl phthalate	DBP	C_16_H_22_O_4_	278.34	84-74-2
Dicyclohexyl phthalate	DCHP	C_20_H_26_O_4_	330.42	84-61-7
Bis-(2-ethylhexyl) phthalate	DEHP	C_24_H_38_O_4_	390.56	117-81-7
Diethyl phthalate	DEP	C_12_H_14_O_4_	222.24	84-66-2
Diisobutyl phthalate	DIBP	C_16_H_22_O_4_	278.34	84-69-5
Diisononyl phthalate	DINP	C_26_H_42_O_4_	418.61	28553-12-0
Dimethyl phthalate	DMP	C_10_H_10_O_4_	194.18	131-11-3
Di-n-octyl phthalate	DNOP	C_24_H_38_O_4_	390.56	117-84-0
Diphenyl phthalate	DphP	C_20_H_14_O_4_	318.32	84-62-8
Dipropyl phthalate	DPP	C_14_H_18_O_4_	250.29	131-16-8
**Non-phthalates**				
Di-(2-ethylhexyl) terephthalate	DEHT	C_24_H_38_O_4_	390.56	6422-86-2
Tri-octyl trimellitate	TOTM	C_33_H_54_O_6_	546.78	3319-31-1

**Table 2:** Acquisition parameters for the GC–MS analysis of plasticizers and internal standards.

Group	Analytes	RT (min)	Quantification Ion (*m*/*z*)	Qualification Ion (*m*/*z*)
1st group from 0 to 13 min	DMP	5.8	163.0	77.0
	DEP	6.6	149.0	177.0
	DEP-d4	6.6	153.0	181.0
	DPP	7.8	149.0	191.1
	DIBP	8.5	149.0	57.1
	DBP	9.2	149.0	223.1
	BBP	12.5	149.0	91.1
2nd group from 13 to 20 min	DCHP	14.6	249.0	149.0
	DEHP	14.8	149.0	279.0
	DphP	15.0	225.0	77.1
	DNOP	18.2	149.0	279.1
	DEHT	18.3	112.1	261.1
	DINP	18.8	293.1	149.0
3rd group from 20 to 35 min	TOTM	31.7	305.1	193.0

RT—retention time.

**Table 3:** Validation parameters (response function, linearity, and LLOQ) for the analysis of plasticizers by GC–MS and HPLC–DAD.

**GC–MS**					
**Analytes**	**Response Function**		**Linearity**		**LLOQ (mg L^−1^)**
	**Model**	**Equation**	**Equation**	**R^2^**	
DMP	1/x weighted linear	12.90x + 1.172 × 10^−2^	0.9422x + 2.761 × 10^−3^	0.994	0.040
DEP	1/x weighted linear	6.456x − 3.294 × 10^−2^	1.001x + 3.113 × 10^−4^	0.997	0.020
DPP	1/x weighted linear	9.337x + 8.978 × 10^−3^	1.007x + 2.870 × 10^−4^	0.991	0.040
DphP	quadratic	1.315x^2^ + 1.377x − 4.667 × 10^−3^	1.004x + 9.521 × 10^−4^	0.98	0.060
BBP	quadratic	0.4008x^2^ + 6.262 × 10^−1^x + 2.761 × 10^−1^	1.018x + 8.234 × 10^−2^	0.994	2.4
DBP	1/x weighted linear	8.154x + 2.770 × 10^−2^	1.022x + 7.642 × 10^−4^	0.99	0.020
DCHP	-	-	-	-	-
DEHP	1/x weighted linear	1.950x − 2.870 × 10^−1^	1.056x − 7.081 × 10^−2^	0.98	0.40
DNOP	-	-	-	-	-
DEHT	1/x weighted linear	0.3805x − 5.397 × 10^−2^	0.993x + 1.280 × 10^−2^	0.98	0.40
DINP	quadratic	0.002715x^2^ + 1.201 × 10^−1^x + 2.067 × 10^−1^	1.005x − 1.200	0.993	40
TOTM	linear	0.09951x − 1.763	1.050x − 1.173	0.98	25
DIBP	1/x weighted linear	7.609x + 1.475 × 10^−2^	0.9671x + 8.764 × 10^−4^	0.97	0.050
**HPLC–DAD**					
**Analytes**	**Response Function**		**Linearity**		**LLOQ (mg L^−1^)**
	**Model**	**Equation**	**Equation**	**R^2^**	
DMP	linear	55,234x − 344	1.007x − 5.54 × 10^−4^	0.999	0.050
DEP	1/x weighted linear	50,225x − 253	1.004x + 3.60 × 10^−3^	0.998	0.050
DPP	1/x weighted linear	44,732x − 243	0.9946x + 4.90 × 10^−3^	0.999	0.050
DphP	linear	51,953x + 7	0.9848x + 5.77 × 10^−3^	0.999	0.050
BBP	linear	39,713x + 78	0.9832x + 4.00 × 10^−3^	0.998	0.050
DBP	linear	40,661x + 274	0.9804x + 5.62 × 10^−3^	0.998	0.050
DCHP	linear	34,991x + 2.5	0.9922x + 2.57 × 10^−3^	0.998	0.050
DEHP	linear	28,433x − 161	0.9877x + 5.30 × 10^−2^	0.996	0.050
DNOP	linear	28,041x + 94	1.010x + 1.60 × 10^−3^	0.998	0.050
DEHT	linear	65,922x + 405	1.011x + 9.90 × 10^−4^	0.997	0.050
DINP	1/x weighted linear	16,323x + 1020	0.9853x + 5.98 × 10^−3^	0.9994	0.50
TOTM	linear	27,046x − 141	1.024x − 5.45 × 10^−3^	0.998	0.050
DIBP	linear	44,105x − 1248	1.020x − 7.49 × 10^−4^	0.9997	0.050

**Table 4:** Amount (%, *w*/*w*) of plasticizers ± standard deviation (three replicates by method) found in five vinyl gloves studied ND: C < LLOQ.

% Plasticizers (*w*/*w*)						
Analytes	Method	V1	V2	V3	V4	V5
DMP	GC–MS	(4.83 ± 0.33) × 10^−4^	(4.67 ± 0.27) × 10^−4^	ND	ND	ND
	HPLC–DAD	ND	ND	ND	ND	ND
DEP	GC–MS	(5.83 ± 0.67) × 10^−4^	(3.00 ± 0.40) × 10^−4^	ND	ND	ND
	HPLC–DAD	ND	ND	ND	ND	ND
DIBP	GC–MS	(9.68 ± 0.79) × 10^−3^	(4.80 ± 0.10) × 10^−3^	(8.00 ± 0.34) × 10^−4^	(1.00 ± 0.34) × 10^−3^	(5.20 ± 0.87) × 10^−4^
	HPLC–DAD	ND	ND	ND	ND	ND
DBP	GC–MS	(4.97 ± 0.40) × 10^−4^	(2.00 ± 0.07) × 10^−3^	(3.33 ± 0.30) × 10^−4^	(1.67 ± 0.28) × 10^−3^	(1.87 ± 0.30) × 10^−3^
	HPLC–DAD	ND	ND	ND	ND	ND
DEHP	GC–MS	1.58 ± 0.09	(8.73 ± 0.03) × 10^−3^	(3.39 ± 0.02) × 10^−1^	4.10 ± 0.41	(6.20 ± 0.66) × 10^−4^
	HPLC–DAD	1.92 ± 0.25	ND	0.297 ± 0.034	3.65 ± 0.64	ND
DEHT	GC–MS	ND	14.9 ± 1.3	(4.77 ± 0.13) × 10^−2^	(3.87 ± 0.72) × 10^−2^	44.4 ± 2.4
	HPLC–DAD	ND	15.6 ± 1.6	ND	ND	36.3 ± 3.7
DINP	GC–MS	35.8 ± 4.5	16.5 ± 0.8	30.2 ± 3.1	35.7 ± 0.5	ND
	HPLC–DAD	40.1 ± 1.2	16.4 ± 1.3	32.9 ± 1.0	35.3 ± 0.9	ND

## Supplementary Materials

The following are available online at https://www.mdpi.com/article/10.3390/toxics9090200/s1, Figure S1: Accuracy profiles of each investigated plasticizer obtained by GC-MS. Figure S2: Accuracy profiles of each investigated plasticizer obtained by HPLC-DAD.
