# Supplementary material for: Analysis of Phthalates and Alternative Plasticizers in Gloves by Gas Chromatography–Mass Spectrometry and Liquid Chromatography–UV Detection: A Comparative Study
Source: Toxics. 2021 Aug 28;9(9):200. doi: 10.3390/toxics9090200 (PMC8472278; doi:10.3390/toxics9090200)
Supplement: Supplementary file 1 [file toxics-09-00200-s001.zip › toxics-1338893-supplementary.pdf]

toxics

MDPI

# Supplementary Materials: Analysis of Phthalates and Alternative Plasticizers in Gloves by Gas Chromatography–Mass Spectrometry and Liquid

# Chromatography–UV Detection: A Comparative Study

**Kelly Poitou, Tiphaine Rogez-Florent, Marie Lecoeur, Cécile Danel, Romain Regnault, Philippe Vérité, Christelle Monteil and Catherine Foulon**

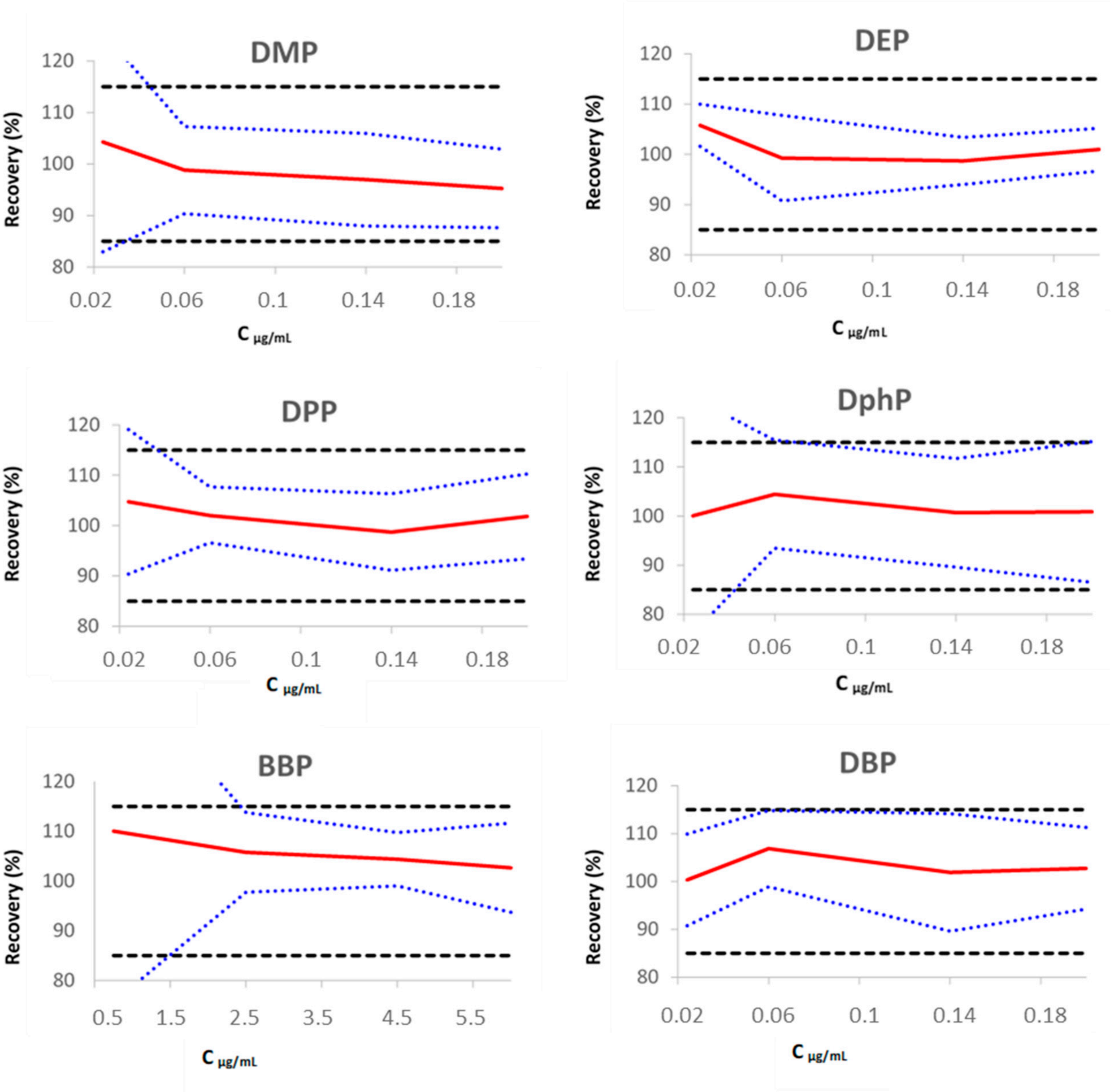

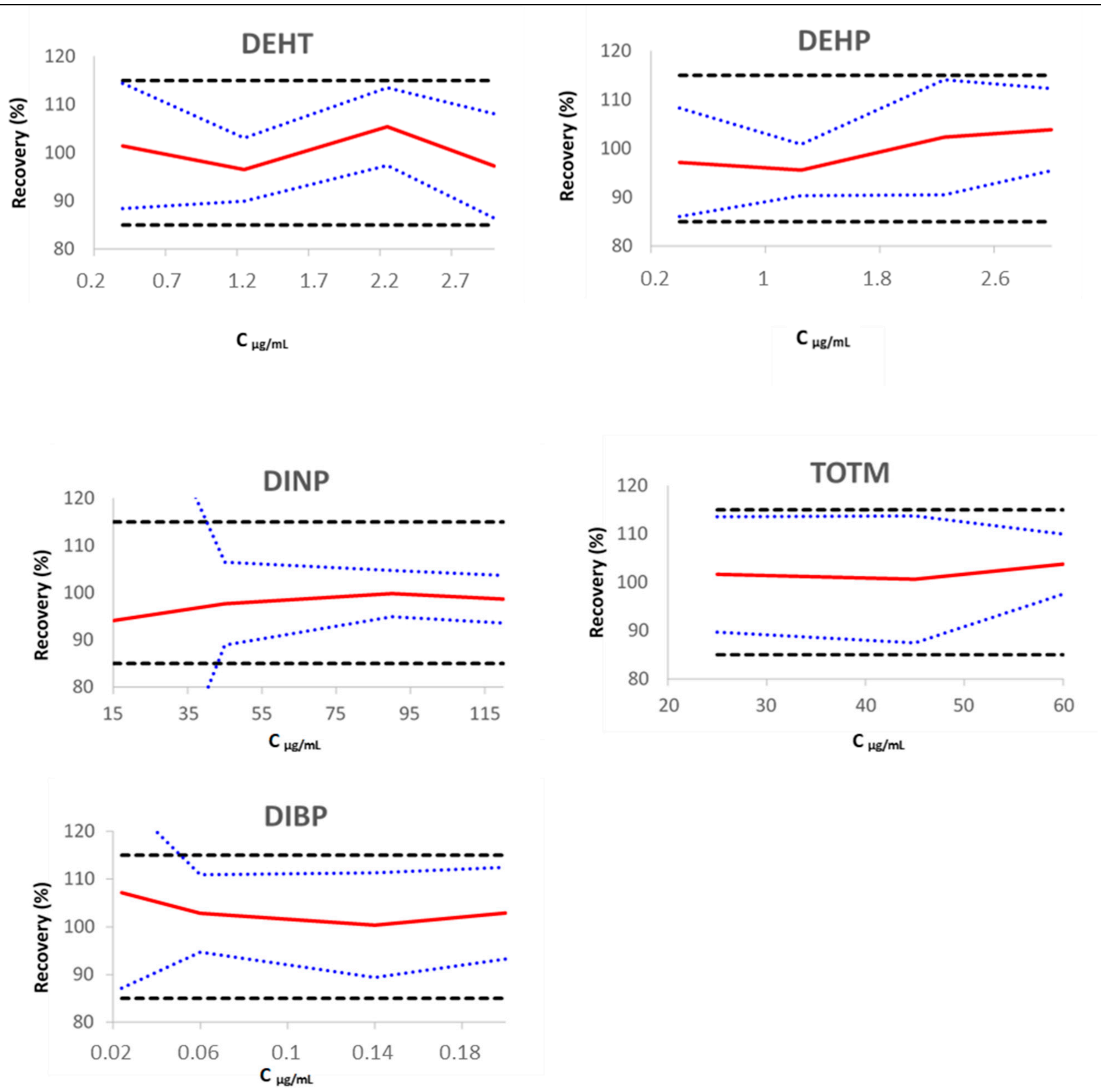


**Figure S1.** Accuracy profiles of each investigated plasticizer obtained by GC-MS. The dashed lines represent the acceptance limits of 15%, the red plain line corresponds to the bias. The tolerance interval of the bias, for a risk of 5%, was materialized by dotted blue lines.

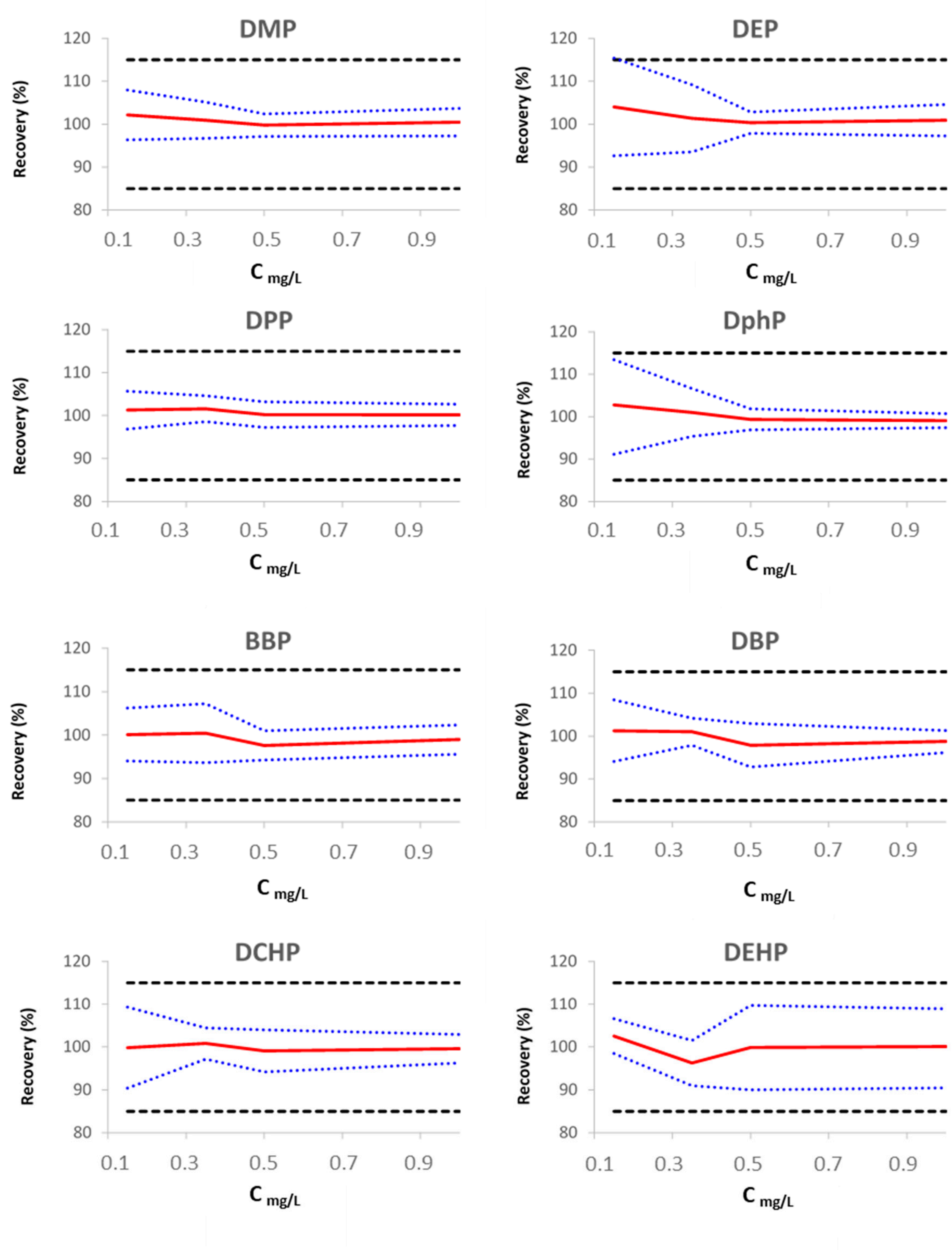
DMP
DEP
DPP
DphP
BBP
DBP
DCHP
DEHP
Recovery (%)
C mg/L
120
110
100
90
80
0.1
0.3
0.5
0.7
0.9

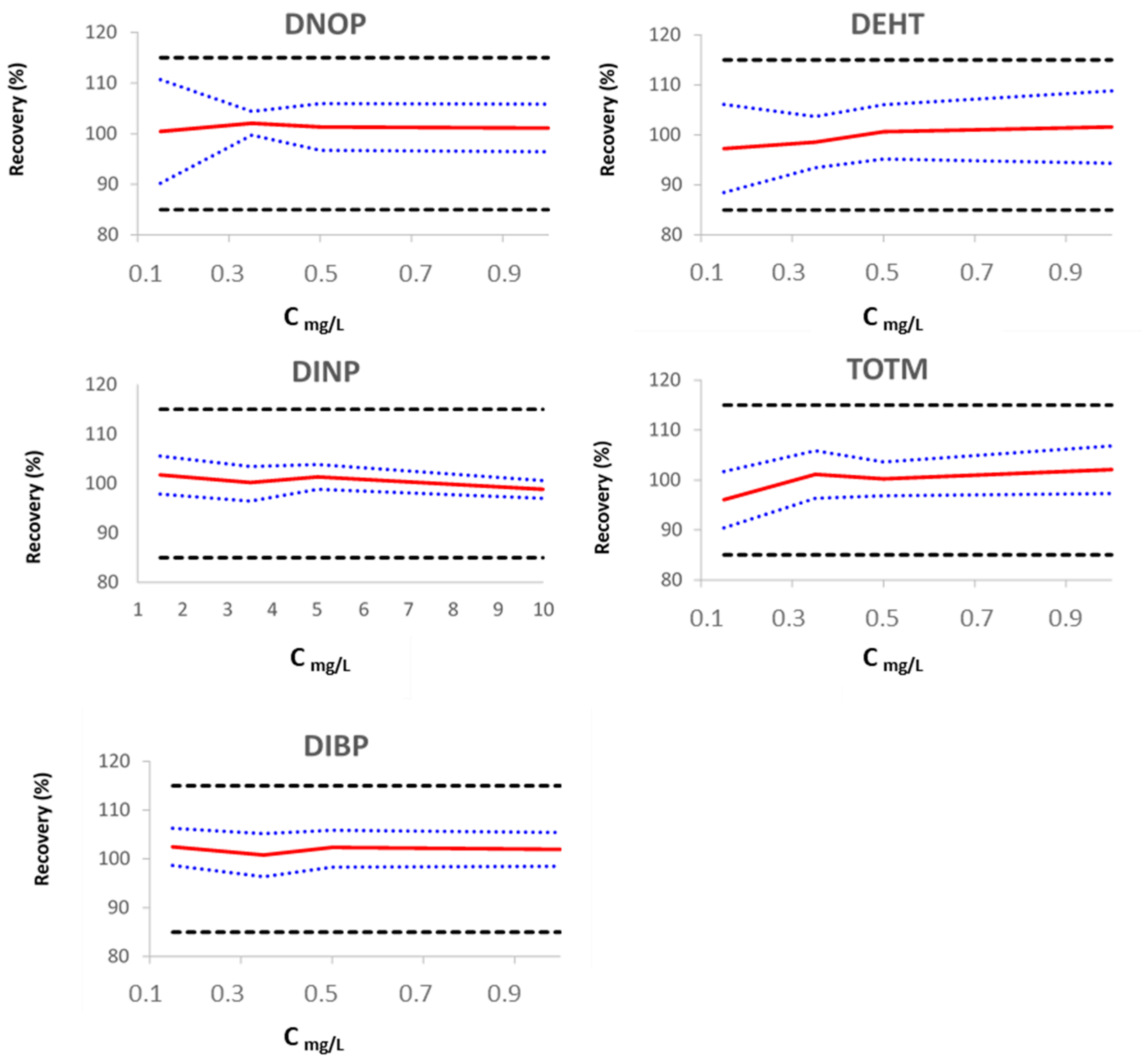


**Figure S2.** Accuracy profiles of each investigated plasticizer obtained by HPLC-DAD. The dashed lines represent the acceptance limits of 15%, the red plain line corresponds to the bias. The tolerance interval of the bias, for a risk of 5%, was materialized by dotted blue lines.
